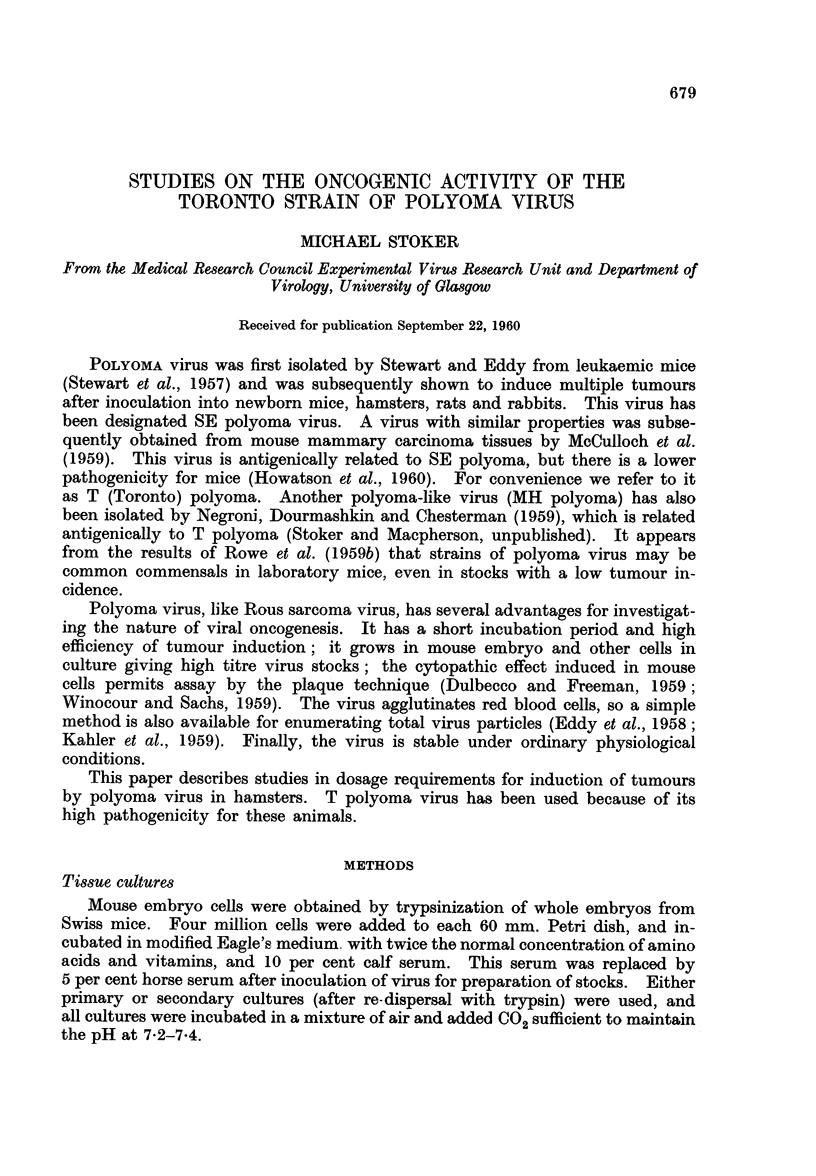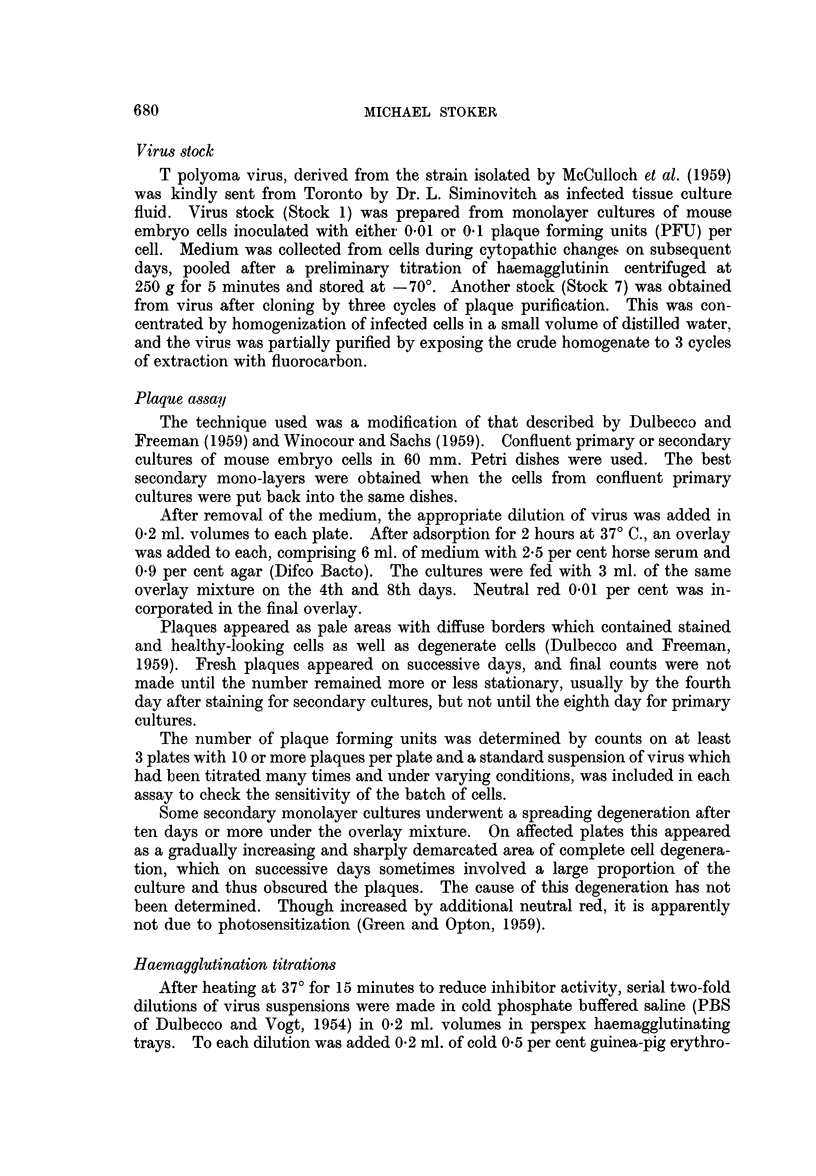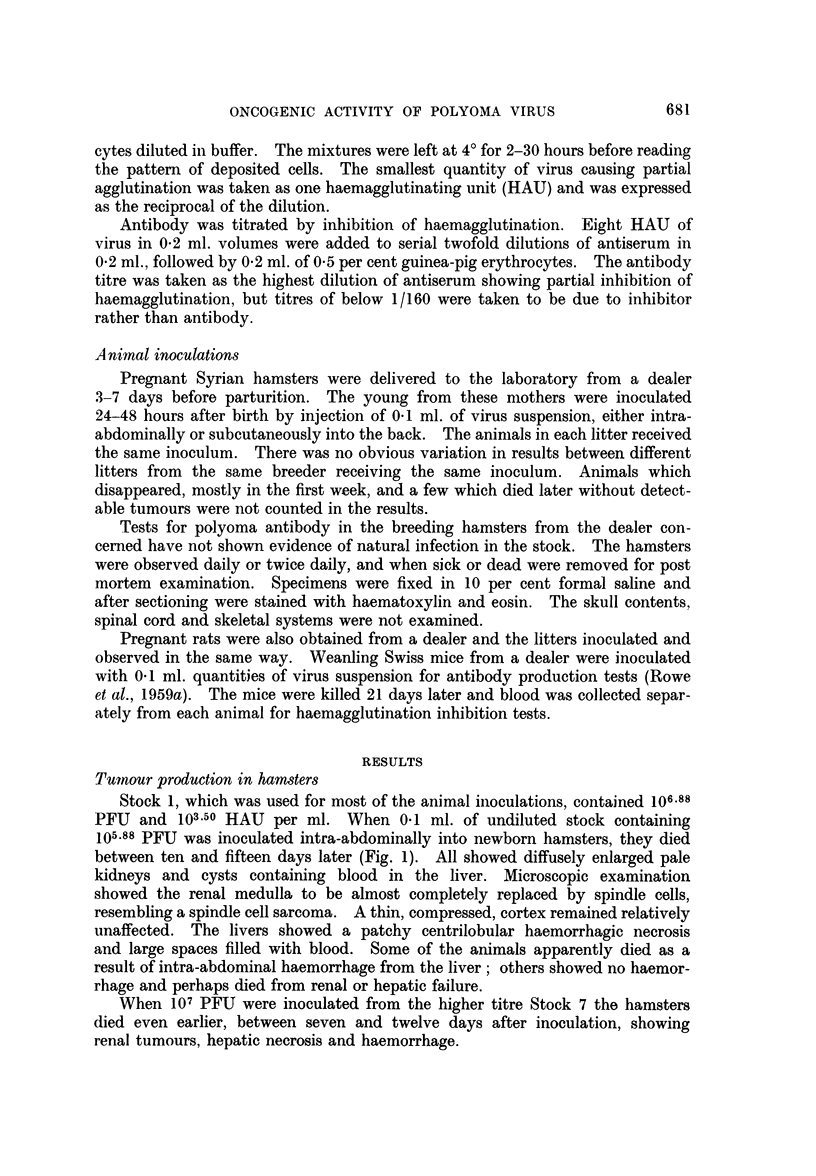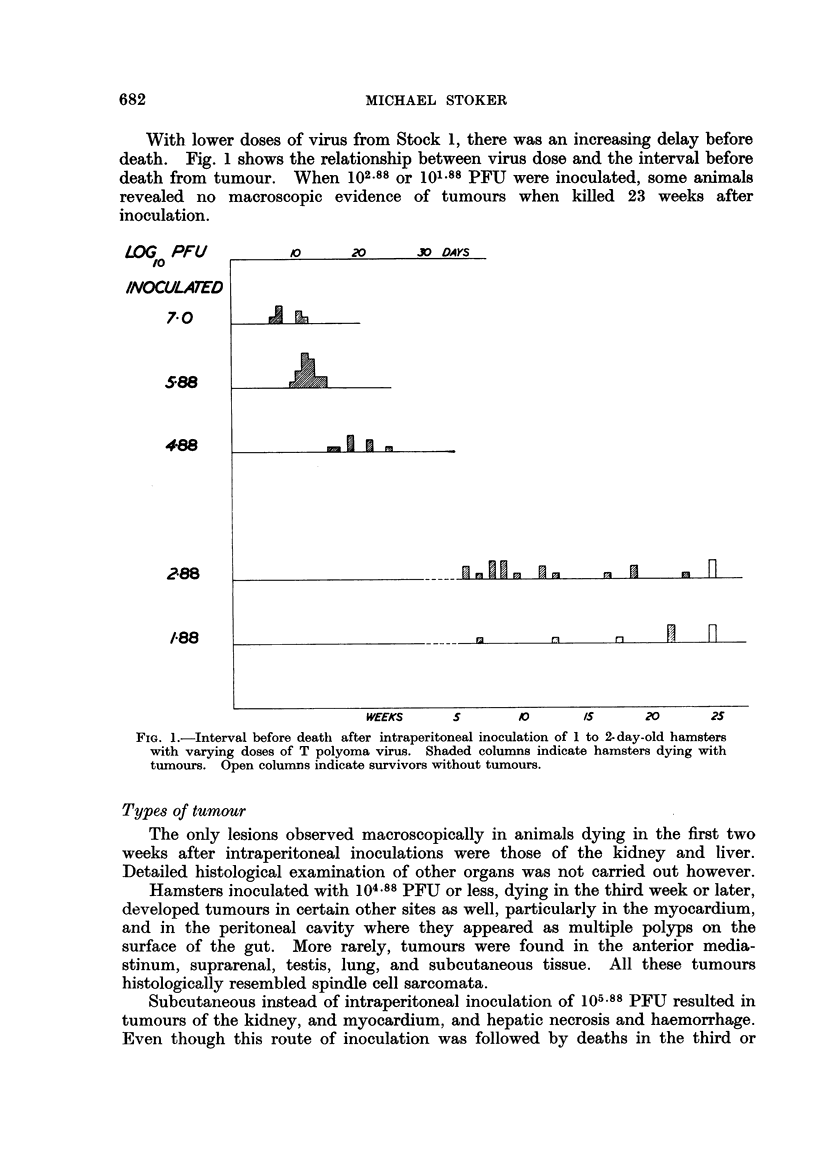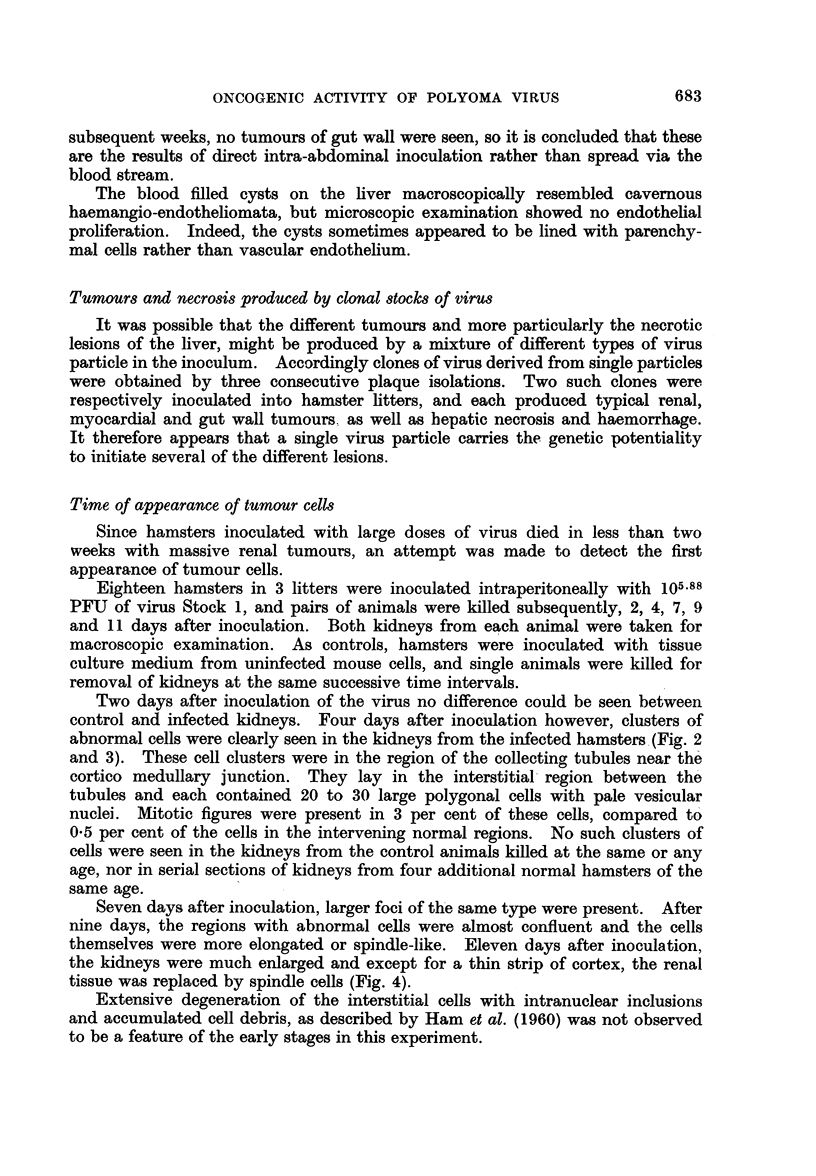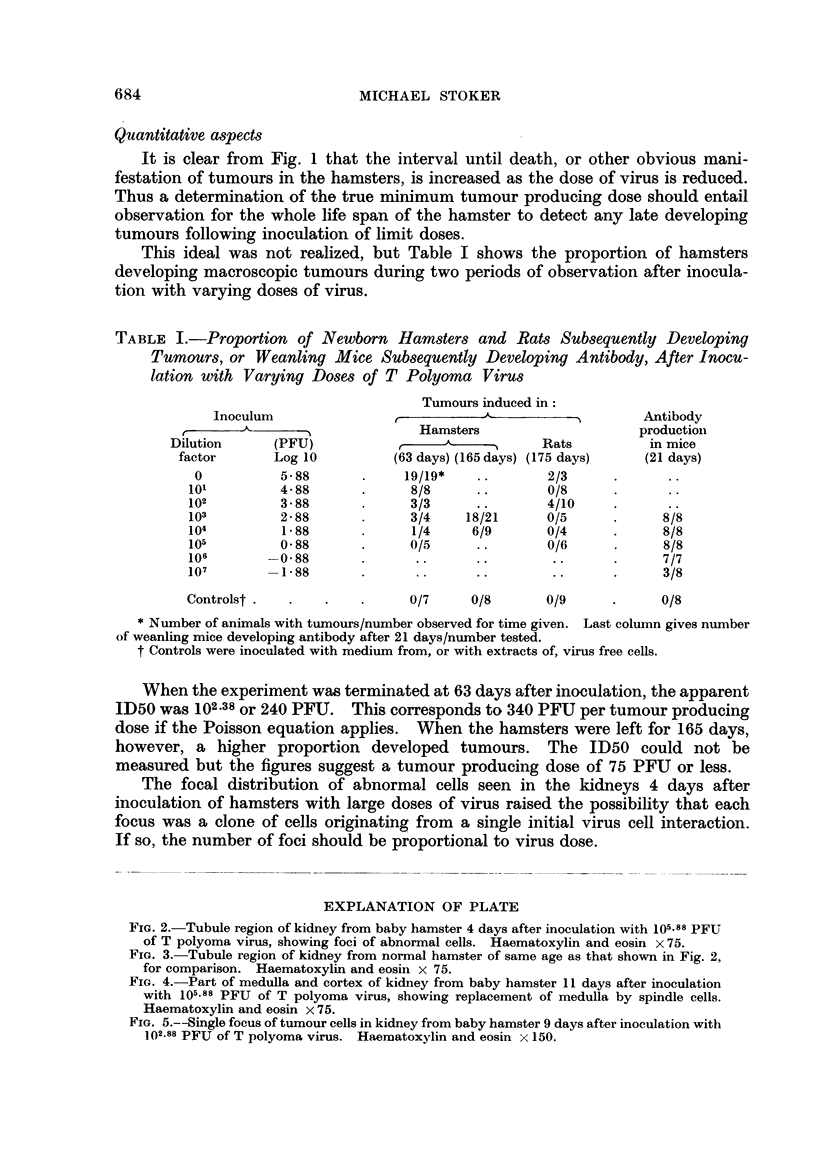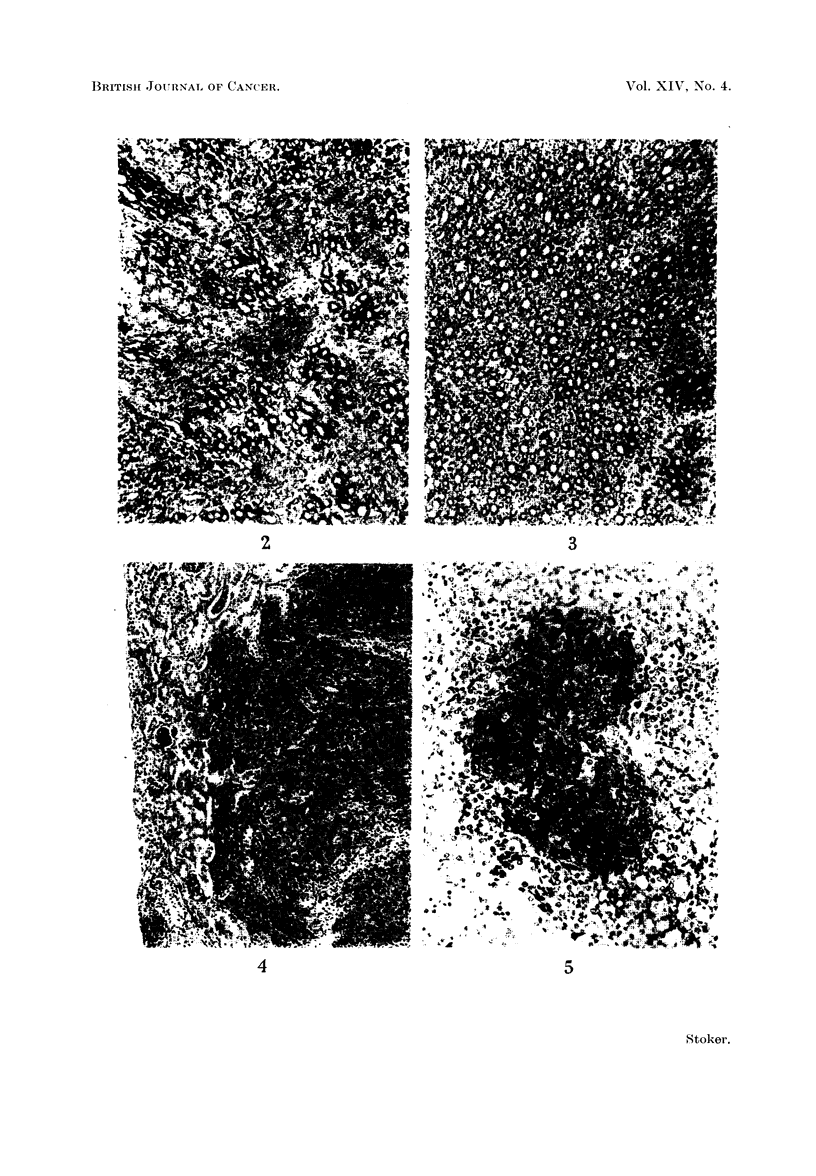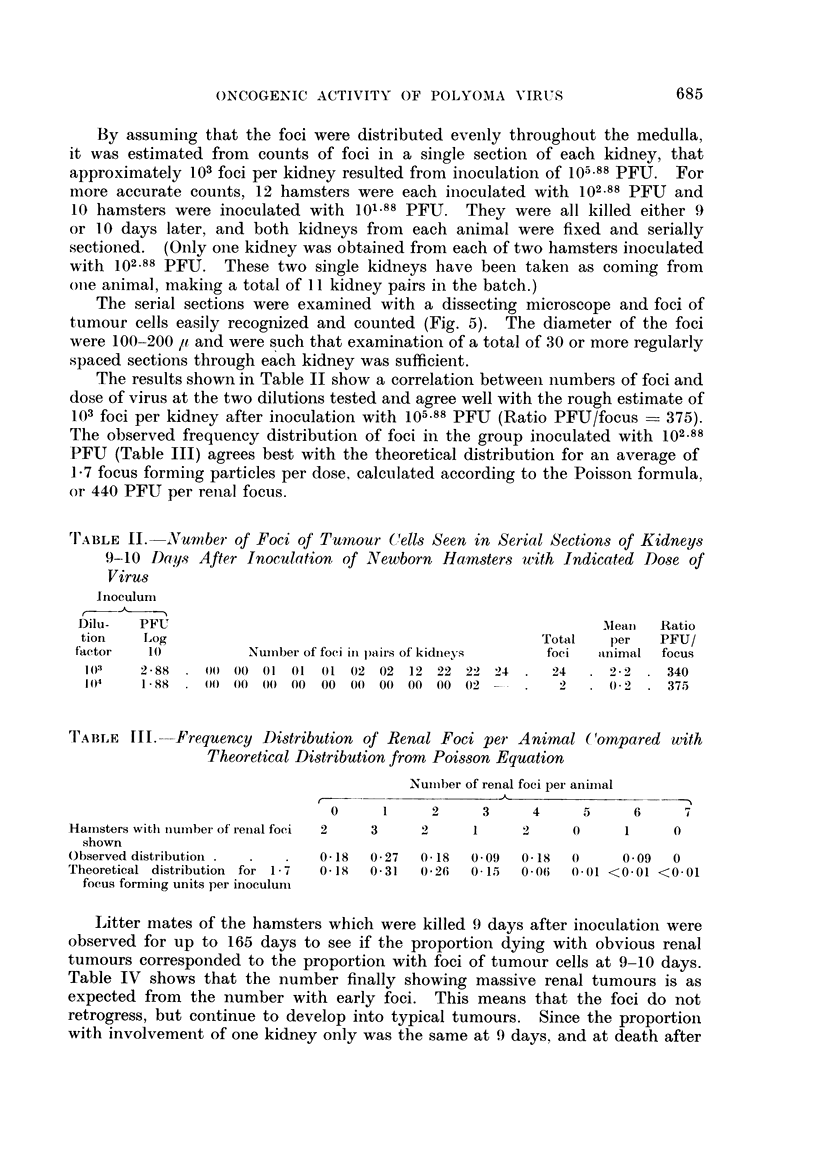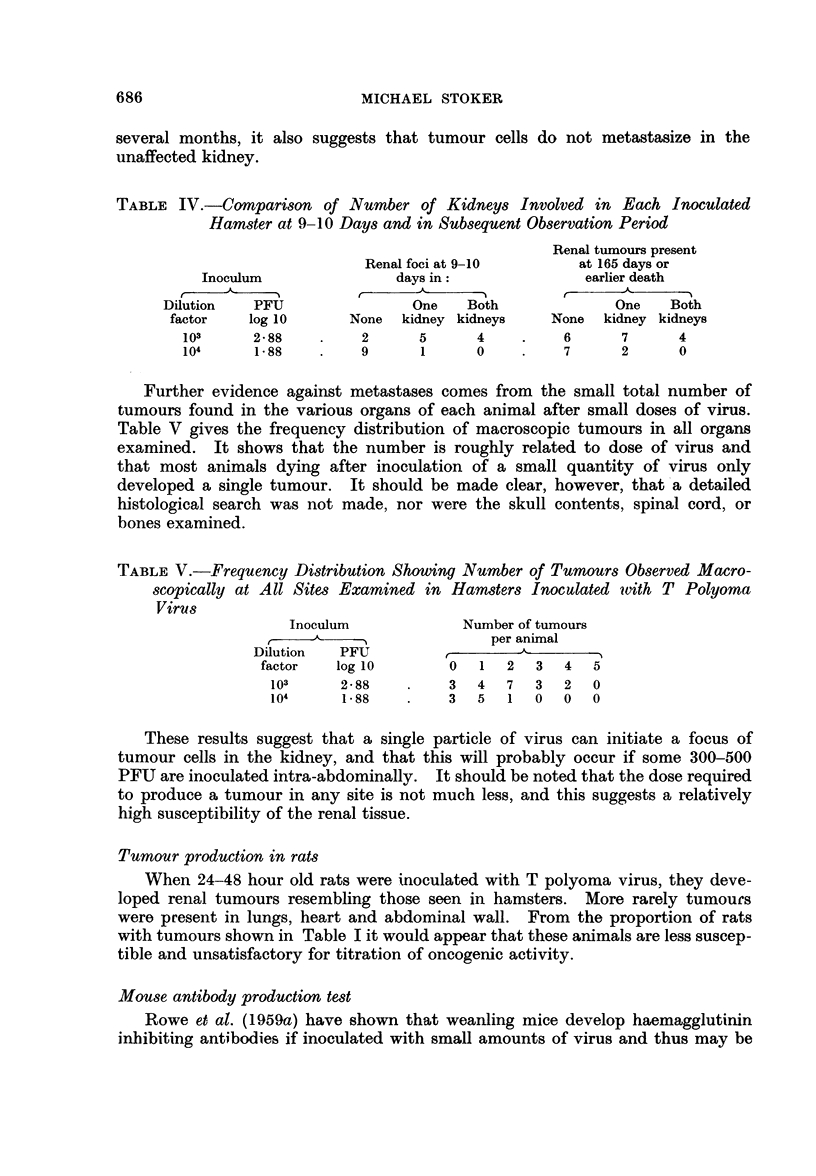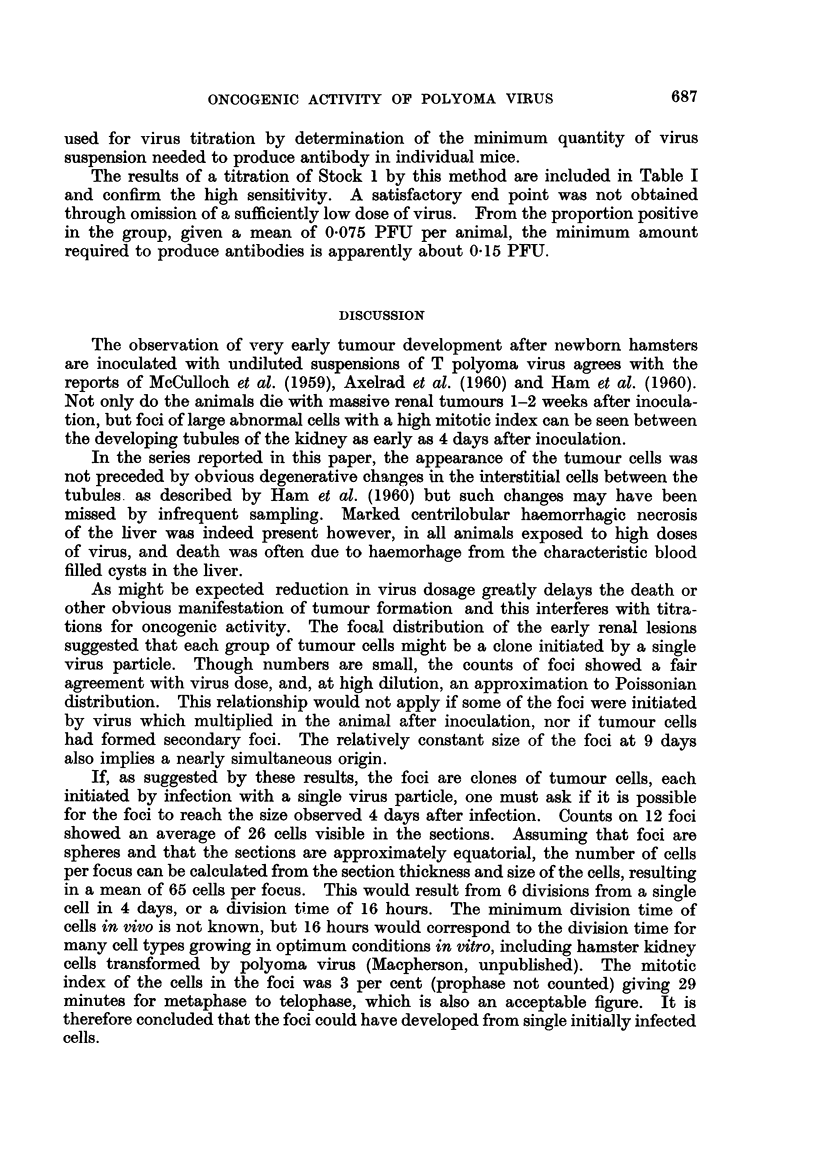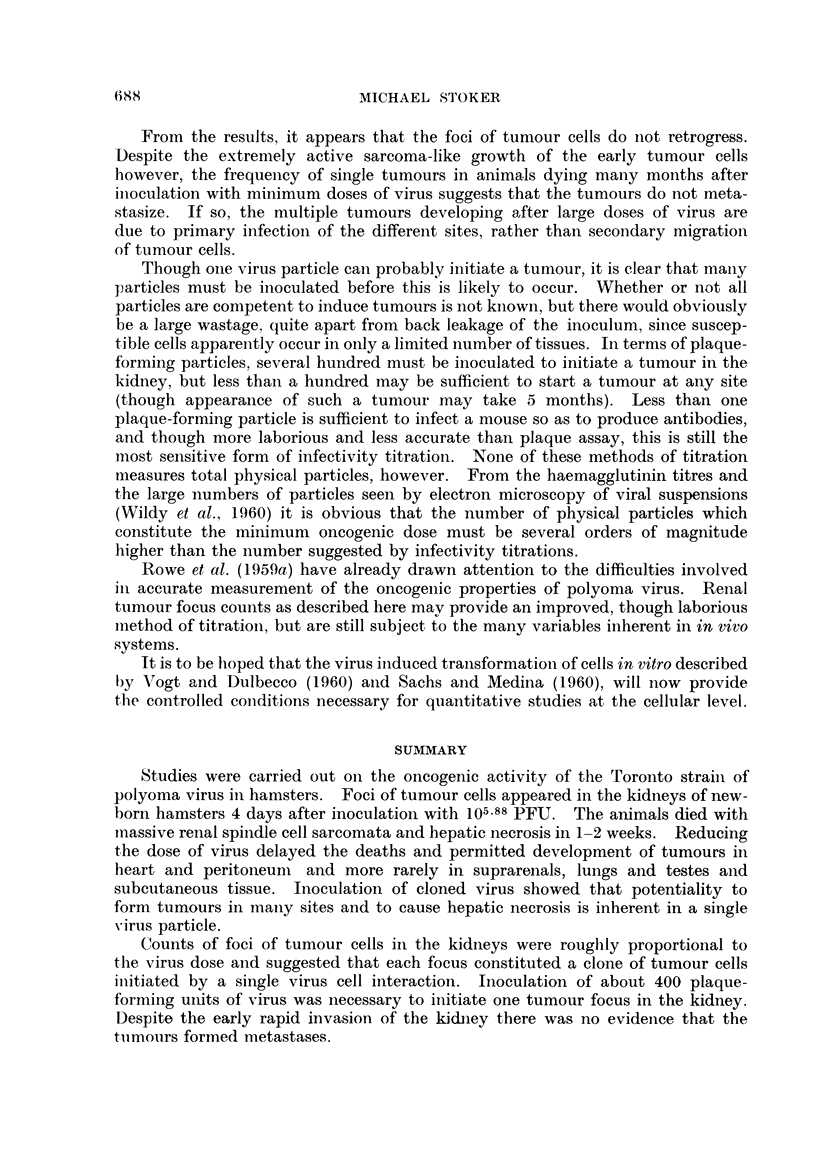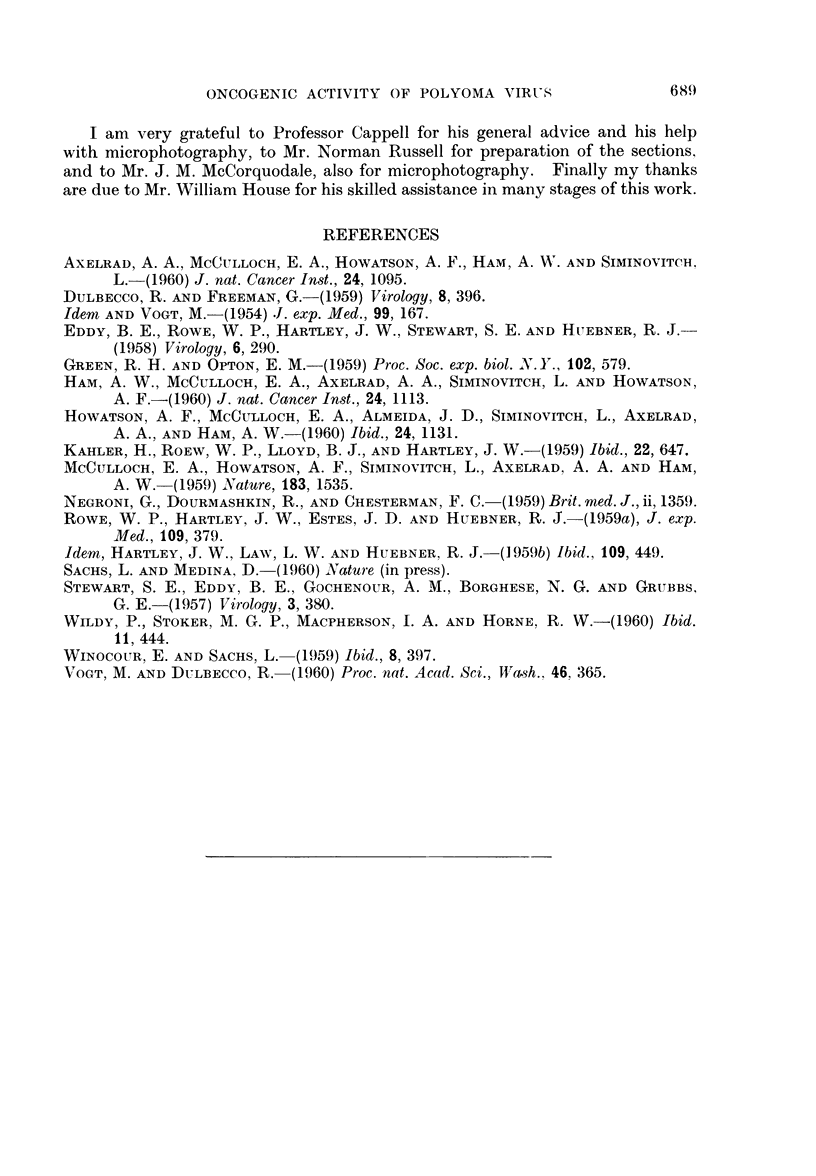# Studies on the Oncogenic Activity of the Toronto Strain of Polyoma Virus

**DOI:** 10.1038/bjc.1960.76

**Published:** 1960-12

**Authors:** Michael Stoker

## Abstract

**Images:**


### STUDIES ON THE ONCOGENIC ACTIVITY OF THE TORONTO STRAIN OF POLYOMA VIRUS

MICHAEL STOKER

*From the Medical Research Council Experimental Virus Research Unit and Department of Virology, University of Glasgow*

Received for publication September 22, 1960

POLYOMA virus was first isolated by Stewart and Eddy from leukaemic mice (Stewart *et al.*, 1957) and was subsequently shown to induce multiple tumours after inoculation into newborn mice, hamsters, rats and rabbits. This virus has been designated SE polyoma virus. A virus with similar properties was subsequently obtained from mouse mammary carcinoma tissues by McCulloch *et al.* (1959). This virus is antigenically related to SE polyoma, but there is a lower pathogenicity for mice (Howatson *et al.*, 1960). For convenience we refer to it as T (Toronto) polyoma. Another polyoma-like virus (MH polyoma) has also been isolated by Negroni, Dourmashkin and Chesterman (1959), which is related antigenically to T polyoma (Stoker and Macpherson, unpublished). It appears from the results of Rowe *et al.* (1959*b*) that strains of polyoma virus may be common commensals in laboratory mice, even in stocks with a low tumour incidence.

Polyoma virus, like Rous sarcoma virus, has several advantages for investigating the nature of viral oncogenesis. It has a short incubation period and high efficiency of tumour induction ; it grows in mouse embryo and other cells in culture giving high titre virus stocks ; the cytopathic effect induced in mouse cells permits assay by the plaque technique (Dulbecco and Freeman, 1959 ; Winocour and Sachs, 1959). The virus agglutinates red blood cells, so a simple method is also available for enumerating total virus particles (Eddy *et al.*, 1958 ; Kahler *et al.*, 1959). Finally, the virus is stable under ordinary physiological conditions.

This paper describes studies in dosage requirements for induction of tumours by polyoma virus in hamsters. T polyoma virus has been used because of its high pathogenicity for these animals.

### METHODS

### *Tissue cultures*

Mouse embryo cells were obtained by trypsinization of whole embryos from Swiss mice. Four million cells were added to each 60 mm. Petri dish, and incubated in modified Eagle's medium with twice the normal concentration of amino acids and vitamins, and 10 per cent calf serum. This serum was replaced by 5 per cent horse serum after inoculation of virus for preparation of stocks. Either primary or secondary cultures (after re-dispersal with trypsin) were used, and all cultures were incubated in a mixture of air and added $CO_2$ sufficient to maintain the pH at 7·2–7·4.

*Virus stock*

T polyoma virus, derived from the strain isolated by McCulloch *et al.* (1959) was kindly sent from Toronto by Dr. L. Siminovitch as infected tissue culture fluid. Virus stock (Stock 1) was prepared from monolayer cultures of mouse embryo cells inoculated with either 0·01 or 0·1 plaque forming units (PFU) per cell. Medium was collected from cells during cytopathic changes on subsequent days, pooled after a preliminary titration of haemagglutinin centrifuged at 250 g for 5 minutes and stored at −70°. Another stock (Stock 7) was obtained from virus after cloning by three cycles of plaque purification. This was concentrated by homogenization of infected cells in a small volume of distilled water, and the virus was partially purified by exposing the crude homogenate to 3 cycles of extraction with fluorocarbon.

*Plaque assay*

The technique used was a modification of that described by Dulbecco and Freeman (1959) and Winocour and Sachs (1959). Confluent primary or secondary cultures of mouse embryo cells in 60 mm. Petri dishes were used. The best secondary mono-layers were obtained when the cells from confluent primary cultures were put back into the same dishes.

After removal of the medium, the appropriate dilution of virus was added in 0·2 ml. volumes to each plate. After adsorption for 2 hours at 37° C., an overlay was added to each, comprising 6 ml. of medium with 2·5 per cent horse serum and 0·9 per cent agar (Difco Bacto). The cultures were fed with 3 ml. of the same overlay mixture on the 4th and 8th days. Neutral red 0·01 per cent was incorporated in the final overlay.

Plaques appeared as pale areas with diffuse borders which contained stained and healthy-looking cells as well as degenerate cells (Dulbecco and Freeman, 1959). Fresh plaques appeared on successive days, and final counts were not made until the number remained more or less stationary, usually by the fourth day after staining for secondary cultures, but not until the eighth day for primary cultures.

The number of plaque forming units was determined by counts on at least 3 plates with 10 or more plaques per plate and a standard suspension of virus which had been titrated many times and under varying conditions, was included in each assay to check the sensitivity of the batch of cells.

Some secondary monolayer cultures underwent a spreading degeneration after ten days or more under the overlay mixture. On affected plates this appeared as a gradually increasing and sharply demarcated area of complete cell degeneration, which on successive days sometimes involved a large proportion of the culture and thus obscured the plaques. The cause of this degeneration has not been determined. Though increased by additional neutral red, it is apparently not due to photosensitization (Green and Opton, 1959).

*Haemagglutination titrations*

After heating at 37° for 15 minutes to reduce inhibitor activity, serial two-fold dilutions of virus suspensions were made in cold phosphate buffered saline (PBS of Dulbecco and Vogt, 1954) in 0·2 ml. volumes in perspex haemagglutinating trays. To each dilution was added 0·2 ml. of cold 0·5 per cent guinea-pig erythrocytes diluted in buffer. The mixtures were left at 4° for 2–30 hours before reading the pattern of deposited cells. The smallest quantity of virus causing partial agglutination was taken as one haemagglutinating unit (HAU) and was expressed as the reciprocal of the dilution.

Antibody was titrated by inhibition of haemagglutination. Eight HAU of virus in 0·2 ml. volumes were added to serial twofold dilutions of antiserum in 0·2 ml., followed by 0·2 ml. of 0·5 per cent guinea-pig erythrocytes. The antibody titre was taken as the highest dilution of antiserum showing partial inhibition of haemagglutination, but titres of below 1/160 were taken to be due to inhibitor rather than antibody.

*Animal inoculations*

Pregnant Syrian hamsters were delivered to the laboratory from a dealer 3–7 days before parturition. The young from these mothers were inoculated 24–48 hours after birth by injection of 0·1 ml. of virus suspension, either intra-abdominally or subcutaneously into the back. The animals in each litter received the same inoculum. There was no obvious variation in results between different litters from the same breeder receiving the same inoculum. Animals which disappeared, mostly in the first week, and a few which died later without detectable tumours were not counted in the results.

Tests for polyoma antibody in the breeding hamsters from the dealer concerned have not shown evidence of natural infection in the stock. The hamsters were observed daily or twice daily, and when sick or dead were removed for post mortem examination. Specimens were fixed in 10 per cent formal saline and after sectioning were stained with haematoxylin and eosin. The skull contents, spinal cord and skeletal systems were not examined.

Pregnant rats were also obtained from a dealer and the litters inoculated and observed in the same way. Weanling Swiss mice from a dealer were inoculated with 0·1 ml. quantities of virus suspension for antibody production tests (Rowe *et al.*, 1959*a*). The mice were killed 21 days later and blood was collected separately from each animal for haemagglutination inhibition tests.

### RESULTS

*Tumour production in hamsters*

Stock 1, which was used for most of the animal inoculations, contained $10^{6.88}$ PFU and $10^{3.50}$ HAU per ml. When 0·1 ml. of undiluted stock containing $10^{5.88}$ PFU was inoculated intra-abdominally into newborn hamsters, they died between ten and fifteen days later (Fig. 1). All showed diffusely enlarged pale kidneys and cysts containing blood in the liver. Microscopic examination showed the renal medulla to be almost completely replaced by spindle cells, resembling a spindle cell sarcoma. A thin, compressed, cortex remained relatively unaffected. The livers showed a patchy centrilobular haemorrhagic necrosis and large spaces filled with blood. Some of the animals apparently died as a result of intra-abdominal haemorrhage from the liver ; others showed no haemorrhage and perhaps died from renal or hepatic failure.

When $10^7$ PFU were inoculated from the higher titre Stock 7 the hamsters died even earlier, between seven and twelve days after inoculation, showing renal tumours, hepatic necrosis and haemorrhage.

With lower doses of virus from Stock 1, there was an increasing delay before death. Fig. 1 shows the relationship between virus dose and the interval before death from tumour. When $10^{2.88}$ or $10^{1.88}$ PFU were inoculated, some animals revealed no macroscopic evidence of tumours when killed 23 weeks after inoculation.

FIG. 1.—Interval before death after intraperitoneal inoculation of 1 to 2-day-old hamsters with varying doses of T polyoma virus. Shaded columns indicate hamsters dying with tumours. Open columns indicate survivors without tumours.

*Types of tumour*

The only lesions observed macroscopically in animals dying in the first two weeks after intraperitoneal inoculations were those of the kidney and liver. Detailed histological examination of other organs was not carried out however.

Hamsters inoculated with $10^{4.88}$ PFU or less, dying in the third week or later, developed tumours in certain other sites as well, particularly in the myocardium, and in the peritoneal cavity where they appeared as multiple polyps on the surface of the gut. More rarely, tumours were found in the anterior mediastinum, suprarenal, testis, lung, and subcutaneous tissue. All these tumours histologically resembled spindle cell sarcomata.

Subcutaneous instead of intraperitoneal inoculation of $10^{5.88}$ PFU resulted in tumours of the kidney, and myocardium, and hepatic necrosis and haemorrhage. Even though this route of inoculation was followed by deaths in the third or subsequent weeks, no tumours of gut wall were seen, so it is concluded that these are the results of direct intra-abdominal inoculation rather than spread via the blood stream.

The blood filled cysts on the liver macroscopically resembled cavernous haemangio-endotheliomata, but microscopic examination showed no endothelial proliferation. Indeed, the cysts sometimes appeared to be lined with parenchymal cells rather than vascular endothelium.

*Tumours and necrosis produced by clonal stocks of virus*

It was possible that the different tumours and more particularly the necrotic lesions of the liver, might be produced by a mixture of different types of virus particle in the inoculum. Accordingly clones of virus derived from single particles were obtained by three consecutive plaque isolations. Two such clones were respectively inoculated into hamster litters, and each produced typical renal, myocardial and gut wall tumours, as well as hepatic necrosis and haemorrhage. It therefore appears that a single virus particle carries the genetic potentiality to initiate several of the different lesions.

*Time of appearance of tumour cells*

Since hamsters inoculated with large doses of virus died in less than two weeks with massive renal tumours, an attempt was made to detect the first appearance of tumour cells.

Eighteen hamsters in 3 litters were inoculated intraperitoneally with $10^{5\cdot88}$ PFU of virus Stock 1, and pairs of animals were killed subsequently, 2, 4, 7, 9 and 11 days after inoculation. Both kidneys from each animal were taken for macroscopic examination. As controls, hamsters were inoculated with tissue culture medium from uninfected mouse cells, and single animals were killed for removal of kidneys at the same successive time intervals.

Two days after inoculation of the virus no difference could be seen between control and infected kidneys. Four days after inoculation however, clusters of abnormal cells were clearly seen in the kidneys from the infected hamsters (Fig. 2 and 3). These cell clusters were in the region of the collecting tubules near the cortico medullary junction. They lay in the interstitial region between the tubules and each contained 20 to 30 large polygonal cells with pale vesicular nuclei. Mitotic figures were present in 3 per cent of these cells, compared to 0·5 per cent of the cells in the intervening normal regions. No such clusters of cells were seen in the kidneys from the control animals killed at the same or any age, nor in serial sections of kidneys from four additional normal hamsters of the same age.

Seven days after inoculation, larger foci of the same type were present. After nine days, the regions with abnormal cells were almost confluent and the cells themselves were more elongated or spindle-like. Eleven days after inoculation, the kidneys were much enlarged and except for a thin strip of cortex, the renal tissue was replaced by spindle cells (Fig. 4).

Extensive degeneration of the interstitial cells with intranuclear inclusions and accumulated cell debris, as described by Ham *et al.* (1960) was not observed to be a feature of the early stages in this experiment.

*Quantitative aspects*

It is clear from Fig. 1 that the interval until death, or other obvious manifestation of tumours in the hamsters, is increased as the dose of virus is reduced. Thus a determination of the true minimum tumour producing dose should entail observation for the whole life span of the hamster to detect any late developing tumours following inoculation of limit doses.

This ideal was not realized, but Table I shows the proportion of hamsters developing macroscopic tumours during two periods of observation after inoculation with varying doses of virus.

TABLE I.—*Proportion of Newborn Hamsters and Rats Subsequently Developing Tumours, or Weanling Mice Subsequently Developing Antibody, After Inoculation with Varying Doses of T Polyoma Virus*

| Inoculum | | Tumours induced in: Hamsters | | Tumours induced in: Rats | Antibody production in mice |
|---|---|---|---|---|---|
| Dilution factor | (PFU) Log 10 | (63 days) | (165 days) | (175 days) | (21 days) |
| 0 | 5·88 | 19/19* | .. | 2/3 | .. |
| $10^{1}$ | 4·88 | 8/8 | .. | 0/8 | .. |
| $10^{2}$ | 3·88 | 3/3 | .. | 4/10 | .. |
| $10^{3}$ | 2·88 | 3/4 | 18/21 | 0/5 | 8/8 |
| $10^{4}$ | 1·88 | 1/4 | 6/9 | 0/4 | 8/8 |
| $10^{5}$ | 0·88 | 0/5 | .. | 0/6 | 8/8 |
| $10^{6}$ | −0·88 | .. | .. | .. | 7/7 |
| $10^{7}$ | −1·88 | .. | .. | .. | 3/8 |
| Controls† | | 0/7 | 0/8 | 0/9 | 0/8 |

* Number of animals with tumours/number observed for time given. Last column gives number of weanling mice developing antibody after 21 days/number tested.

† Controls were inoculated with medium from, or with extracts of, virus free cells.

When the experiment was terminated at 63 days after inoculation, the apparent ID50 was $10^{2.38}$ or 240 PFU. This corresponds to 340 PFU per tumour producing dose if the Poisson equation applies. When the hamsters were left for 165 days, however, a higher proportion developed tumours. The ID50 could not be measured but the figures suggest a tumour producing dose of 75 PFU or less.

The focal distribution of abnormal cells seen in the kidneys 4 days after inoculation of hamsters with large doses of virus raised the possibility that each focus was a clone of cells originating from a single initial virus cell interaction. If so, the number of foci should be proportional to virus dose.

### EXPLANATION OF PLATE

FIG. 2.—Tubule region of kidney from baby hamster 4 days after inoculation with $10^{5.88}$ PFU of T polyoma virus, showing foci of abnormal cells. Haematoxylin and eosin ×75.

FIG. 3.—Tubule region of kidney from normal hamster of same age as that shown in Fig. 2, for comparison. Haematoxylin and eosin × 75.

FIG. 4.—Part of medulla and cortex of kidney from baby hamster 11 days after inoculation with $10^{5.88}$ PFU of T polyoma virus, showing replacement of medulla by spindle cells. Haematoxylin and eosin ×75.

FIG. 5.—Single focus of tumour cells in kidney from baby hamster 9 days after inoculation with $10^{2.88}$ PFU of T polyoma virus. Haematoxylin and eosin ×150.

2

3

4

5

Stoker.

By assuming that the foci were distributed evenly throughout the medulla, it was estimated from counts of foci in a single section of each kidney, that approximately $10^3$ foci per kidney resulted from inoculation of $10^{5 \cdot 88}$ PFU. For more accurate counts, 12 hamsters were each inoculated with $10^{2 \cdot 88}$ PFU and 10 hamsters were inoculated with $10^{1 \cdot 88}$ PFU. They were all killed either 9 or 10 days later, and both kidneys from each animal were fixed and serially sectioned. (Only one kidney was obtained from each of two hamsters inoculated with $10^{2 \cdot 88}$ PFU. These two single kidneys have been taken as coming from one animal, making a total of 11 kidney pairs in the batch.)

The serial sections were examined with a dissecting microscope and foci of tumour cells easily recognized and counted (Fig. 5). The diameter of the foci were 100–200 $\mu$ and were such that examination of a total of 30 or more regularly spaced sections through each kidney was sufficient.

The results shown in Table II show a correlation between numbers of foci and dose of virus at the two dilutions tested and agree well with the rough estimate of $10^3$ foci per kidney after inoculation with $10^{5 \cdot 88}$ PFU (Ratio PFU/focus = 375). The observed frequency distribution of foci in the group inoculated with $10^{2 \cdot 88}$ PFU (Table III) agrees best with the theoretical distribution for an average of 1·7 focus forming particles per dose, calculated according to the Poisson formula, or 440 PFU per renal focus.

TABLE II.—*Number of Foci of Tumour Cells Seen in Serial Sections of Kidneys 9–10 Days After Inoculation of Newborn Hamsters with Indicated Dose of Virus*

| Inoculum: Dilution factor | Inoculum: PFU Log 10 | Number of foci in pairs of kidneys | | | | | | | | | | | Total foci | Mean per animal | Ratio PFU/focus |
|---|---|---|---|---|---|---|---|---|---|---|---|---|---|---|---|
| $10^3$ | 2·88 | 00 | 00 | 01 | 01 | 01 | 02 | 02 | 12 | 22 | 22 | 24 | 24 | 2·2 | 340 |
| $10^4$ | 1·88 | 00 | 00 | 00 | 00 | 00 | 00 | 00 | 00 | 00 | 02 | — | 2 | 0·2 | 375 |

TABLE III.—*Frequency Distribution of Renal Foci per Animal Compared with Theoretical Distribution from Poisson Equation*

| | Number of renal foci per animal: 0 | 1 | 2 | 3 | 4 | 5 | 6 | 7 |
|---|---|---|---|---|---|---|---|---|
| Hamsters with number of renal foci shown | 2 | 3 | 2 | 1 | 2 | 0 | 1 | 0 |
| Observed distribution . . . | 0·18 | 0·27 | 0·18 | 0·09 | 0·18 | 0 | 0·09 | 0 |
| Theoretical distribution for 1·7 focus forming units per inoculum | 0·18 | 0·31 | 0·26 | 0·15 | 0·06 | 0·01 | $<0 \cdot 01$ | $<0 \cdot 01$ |

Litter mates of the hamsters which were killed 9 days after inoculation were observed for up to 165 days to see if the proportion dying with obvious renal tumours corresponded to the proportion with foci of tumour cells at 9–10 days. Table IV shows that the number finally showing massive renal tumours is as expected from the number with early foci. This means that the foci do not retrogress, but continue to develop into typical tumours. Since the proportion with involvement of one kidney only was the same at 9 days, and at death after several months, it also suggests that tumour cells do not metastasize in the unaffected kidney.

TABLE IV.—*Comparison of Number of Kidneys Involved in Each Inoculated Hamster at* 9–10 *Days and in Subsequent Observation Period*

| Inoculum | | | Renal foci at 9–10 days in : | | | | Renal tumours present at 165 days or earlier death | | |
|---|---|---|---|---|---|---|---|---|---|
| Dilution factor | PFU log 10 | | None | One kidney | Both kidneys | | None | One kidney | Both kidneys |
| $10^3$ | 2·88 | . | 2 | 5 | 4 | . | 6 | 7 | 4 |
| $10^4$ | 1·88 | . | 9 | 1 | 0 | . | 7 | 2 | 0 |

Further evidence against metastases comes from the small total number of tumours found in the various organs of each animal after small doses of virus. Table V gives the frequency distribution of macroscopic tumours in all organs examined. It shows that the number is roughly related to dose of virus and that most animals dying after inoculation of a small quantity of virus only developed a single tumour. It should be made clear, however, that a detailed histological search was not made, nor were the skull contents, spinal cord, or bones examined.

TABLE V.—*Frequency Distribution Showing Number of Tumours Observed Macroscopically at All Sites Examined in Hamsters Inoculated with T Polyoma Virus*

| Inoculum | | | Number of tumours per animal | | | | | |
|---|---|---|---|---|---|---|---|---|
| Dilution factor | PFU log 10 | | 0 | 1 | 2 | 3 | 4 | 5 |
| $10^3$ | 2·88 | . | 3 | 4 | 7 | 3 | 2 | 0 |
| $10^4$ | 1·88 | . | 3 | 5 | 1 | 0 | 0 | 0 |

These results suggest that a single particle of virus can initiate a focus of tumour cells in the kidney, and that this will probably occur if some 300–500 PFU are inoculated intra-abdominally. It should be noted that the dose required to produce a tumour in any site is not much less, and this suggests a relatively high susceptibility of the renal tissue.

*Tumour production in rats*

When 24–48 hour old rats were inoculated with T polyoma virus, they developed renal tumours resembling those seen in hamsters. More rarely tumours were present in lungs, heart and abdominal wall. From the proportion of rats with tumours shown in Table I it would appear that these animals are less susceptible and unsatisfactory for titration of oncogenic activity.

*Mouse antibody production test*

Rowe *et al.* (1959*a*) have shown that weanling mice develop haemagglutinin inhibiting antibodies if inoculated with small amounts of virus and thus may be used for virus titration by determination of the minimum quantity of virus suspension needed to produce antibody in individual mice.

The results of a titration of Stock 1 by this method are included in Table I and confirm the high sensitivity. A satisfactory end point was not obtained through omission of a sufficiently low dose of virus. From the proportion positive in the group, given a mean of 0·075 PFU per animal, the minimum amount required to produce antibodies is apparently about 0·15 PFU.

### DISCUSSION

The observation of very early tumour development after newborn hamsters are inoculated with undiluted suspensions of T polyoma virus agrees with the reports of McCulloch *et al.* (1959), Axelrad *et al.* (1960) and Ham *et al.* (1960). Not only do the animals die with massive renal tumours 1–2 weeks after inoculation, but foci of large abnormal cells with a high mitotic index can be seen between the developing tubules of the kidney as early as 4 days after inoculation.

In the series reported in this paper, the appearance of the tumour cells was not preceded by obvious degenerative changes in the interstitial cells between the tubules as described by Ham *et al.* (1960) but such changes may have been missed by infrequent sampling. Marked centrilobular haemorrhagic necrosis of the liver was indeed present however, in all animals exposed to high doses of virus, and death was often due to haemorhage from the characteristic blood filled cysts in the liver.

As might be expected reduction in virus dosage greatly delays the death or other obvious manifestation of tumour formation and this interferes with titrations for oncogenic activity. The focal distribution of the early renal lesions suggested that each group of tumour cells might be a clone initiated by a single virus particle. Though numbers are small, the counts of foci showed a fair agreement with virus dose, and, at high dilution, an approximation to Poissonian distribution. This relationship would not apply if some of the foci were initiated by virus which multiplied in the animal after inoculation, nor if tumour cells had formed secondary foci. The relatively constant size of the foci at 9 days also implies a nearly simultaneous origin.

If, as suggested by these results, the foci are clones of tumour cells, each initiated by infection with a single virus particle, one must ask if it is possible for the foci to reach the size observed 4 days after infection. Counts on 12 foci showed an average of 26 cells visible in the sections. Assuming that foci are spheres and that the sections are approximately equatorial, the number of cells per focus can be calculated from the section thickness and size of the cells, resulting in a mean of 65 cells per focus. This would result from 6 divisions from a single cell in 4 days, or a division time of 16 hours. The minimum division time of cells *in vivo* is not known, but 16 hours would correspond to the division time for many cell types growing in optimum conditions *in vitro*, including hamster kidney cells transformed by polyoma virus (Macpherson, unpublished). The mitotic index of the cells in the foci was 3 per cent (prophase not counted) giving 29 minutes for metaphase to telophase, which is also an acceptable figure. It is therefore concluded that the foci could have developed from single initially infected cells.

From the results, it appears that the foci of tumour cells do not retrogress. Despite the extremely active sarcoma-like growth of the early tumour cells however, the frequency of single tumours in animals dying many months after inoculation with minimum doses of virus suggests that the tumours do not metastasize. If so, the multiple tumours developing after large doses of virus are due to primary infection of the different sites, rather than secondary migration of tumour cells.

Though one virus particle can probably initiate a tumour, it is clear that many particles must be inoculated before this is likely to occur. Whether or not all particles are competent to induce tumours is not known, but there would obviously be a large wastage, quite apart from back leakage of the inoculum, since susceptible cells apparently occur in only a limited number of tissues. In terms of plaque-forming particles, several hundred must be inoculated to initiate a tumour in the kidney, but less than a hundred may be sufficient to start a tumour at any site (though appearance of such a tumour may take 5 months). Less than one plaque-forming particle is sufficient to infect a mouse so as to produce antibodies, and though more laborious and less accurate than plaque assay, this is still the most sensitive form of infectivity titration. None of these methods of titration measures total physical particles, however. From the haemagglutinin titres and the large numbers of particles seen by electron microscopy of viral suspensions (Wildy *et al.*, 1960) it is obvious that the number of physical particles which constitute the minimum oncogenic dose must be several orders of magnitude higher than the number suggested by infectivity titrations.

Rowe *et al.* (1959*a*) have already drawn attention to the difficulties involved in accurate measurement of the oncogenic properties of polyoma virus. Renal tumour focus counts as described here may provide an improved, though laborious method of titration, but are still subject to the many variables inherent in *in vivo* systems.

It is to be hoped that the virus induced transformation of cells *in vitro* described by Vogt and Dulbecco (1960) and Sachs and Medina (1960), will now provide the controlled conditions necessary for quantitative studies at the cellular level.

### SUMMARY

Studies were carried out on the oncogenic activity of the Toronto strain of polyoma virus in hamsters. Foci of tumour cells appeared in the kidneys of new-born hamsters 4 days after inoculation with $10^{5 \cdot 88}$ PFU. The animals died with massive renal spindle cell sarcomata and hepatic necrosis in 1–2 weeks. Reducing the dose of virus delayed the deaths and permitted development of tumours in heart and peritoneum and more rarely in suprarenals, lungs and testes and subcutaneous tissue. Inoculation of cloned virus showed that potentiality to form tumours in many sites and to cause hepatic necrosis is inherent in a single virus particle.

Counts of foci of tumour cells in the kidneys were roughly proportional to the virus dose and suggested that each focus constituted a clone of tumour cells initiated by a single virus cell interaction. Inoculation of about 400 plaque-forming units of virus was necessary to initiate one tumour focus in the kidney. Despite the early rapid invasion of the kidney there was no evidence that the tumours formed metastases.

I am very grateful to Professor Cappell for his general advice and his help with microphotography, to Mr. Norman Russell for preparation of the sections, and to Mr. J. M. McCorquodale, also for microphotography. Finally my thanks are due to Mr. William House for his skilled assistance in many stages of this work.

### REFERENCES

AXELRAD, A. A., McCULLOCH, E. A., HOWATSON, A. F., HAM, A. W. AND SIMINOVITCH, L.—(1960) *J. nat. Cancer Inst.*, **24**, 1095.

DULBECCO, R. AND FREEMAN, G.—(1959) *Virology*, **8**, 396.

*Idem* AND VOGT, M.—(1954) *J. exp. Med.*, **99**, 167.

EDDY, B. E., ROWE, W. P., HARTLEY, J. W., STEWART, S. E. AND HUEBNER, R. J.—(1958) *Virology*, **6**, 290.

GREEN, R. H. AND OPTON, E. M.—(1959) *Proc. Soc. exp. biol. N.Y.*, **102**, 579.

HAM, A. W., McCULLOCH, E. A., AXELRAD, A. A., SIMINOVITCH, L. AND HOWATSON, A. F.—(1960) *J. nat. Cancer Inst.*, **24**, 1113.

HOWATSON, A. F., McCULLOCH, E. A., ALMEIDA, J. D., SIMINOVITCH, L., AXELRAD, A. A., AND HAM, A. W.—(1960) *Ibid.*, **24**, 1131.

KAHLER, H., ROEW, W. P., LLOYD, B. J., AND HARTLEY, J. W.—(1959) *Ibid.*, **22**, 647.

McCULLOCH, E. A., HOWATSON, A. F., SIMINOVITCH, L., AXELRAD, A. A. AND HAM, A. W.—(1959) *Nature*, **183**, 1535.

NEGRONI, G., DOURMASHKIN, R., AND CHESTERMAN, F. C.—(1959) *Brit. med. J.*, ii, 1359.

ROWE, W. P., HARTLEY, J. W., ESTES, J. D. AND HUEBNER, R. J.—(1959*a*), *J. exp. Med.*, **109**, 379.

*Idem*, HARTLEY, J. W., LAW, L. W. AND HUEBNER, R. J.—(1959*b*) *Ibid.*, **109**, 449.

SACHS, L. AND MEDINA, D.—(1960) *Nature* (in press).

STEWART, S. E., EDDY, B. E., GOCHENOUR, A. M., BORGHESE, N. G. AND GRUBBS, G. E.—(1957) *Virology*, **3**, 380.

WILDY, P., STOKER, M. G. P., MACPHERSON, I. A. AND HORNE, R. W.—(1960) *Ibid.* **11**, 444.

WINOCOUR, E. AND SACHS, L.—(1959) *Ibid.*, **8**, 397.

VOGT, M. AND DULBECCO, R.—(1960) *Proc. nat. Acad. Sci., Wash.*, **46**, 365.